# Health effects of reduced occupational sedentary behaviour in type 2 diabetes using a mobile health intervention: a study protocol for a 12-month randomized controlled trial—the ROSEBUD study

**DOI:** 10.1186/s13063-022-06528-x

**Published:** 2022-07-27

**Authors:** M. B. Syrjälä, L. Bennet, P. C. Dempsey, E. Fharm, M. Hellgren, S. Jansson, S. Nilsson, M. Nordendahl, O. Rolandsson, K. Rådholm, A. Ugarph-Morawski, P. Wändell, P. Wennberg

**Affiliations:** 1 Department of Public Health and Clinical Medicine, Family Medicine, Umeå University, Umeå, Sweden; 2 Department of Clinical Sciences, Lund University, Malmö, Sweden; 3 Center for Primary Health Care Research, Region Skåne and Lund University, Malmö, Sweden; 4 Clinical Research and Trial Center, Lund University Hospital, Lund, Sweden; 5 Baker Heart and Diabetes Institute, Melbourne, Australia; 6 MRC Epidemiology Unit, Institute of Metabolic Science, University of Cambridge, Cambridge, UK; 7 Diabetes Research Centre, University of Leicester, Leicester General Hospital, Leicester, UK; 8 Skaraborg Institute, Skövde, Sweden; 9 School of Medical Sciences, University Health Care Research Center, Örebro University, Örebro, Sweden; 10 Department of Public Health and Caring Sciences, Uppsala University, Uppsala, Sweden; 11 Department of Health, Medicine and Caring Sciences, Linköping University, Linköping, Sweden; 12 The George Institute for Global Health, University of New South Wales, Sydney, Australia; 13 Academic Primary Care Center, Region Stockholm, Stockholm, Sweden; 14 Department of Neurobiology, Care Sciences, and Society, Division of Family Medicine and Primary Care, The Karolinska Institute, Huddinge, Sweden

**Keywords:** Type 2 diabetes, Occupational sitting, mHealth, Interventions, Sedentary behaviour, Physical activity, Randomized controlled trial, Workplace, Accelerometer, Behaviour change

## Abstract

**Background:**

Short-term trials conducted in adults with type 2 diabetes mellitus (T2DM) showed that reducing sedentary behaviour by performing regular short bouts of light-intensity physical activity enhances health. Moreover, support for reducing sedentary behaviour may be provided at a low cost via mobile health technology (mHealth). There are a wide range of mHealth solutions available including SMS text message reminders and activity trackers that monitor the physical activity level and notify the user of prolonged sitting periods. The aim of this study is to evaluate the effects of a mHealth intervention on sedentary behaviour and physical activity and the associated changes in health in adults with T2DM.

**Methods:**

A dual-arm, 12-month, randomized controlled trial (RCT) will be conducted within a nationwide Swedish collaboration for diabetes research in primary health care. Individuals with T2DM (*n* = 142) and mainly sedentary work will be recruited across primary health care centres in five regions in Sweden. Participants will be randomized (1:1) into two groups. A mHealth intervention group who will receive an activity tracker wristband (Garmin Vivofit4), regular SMS text message reminders, and counselling with a diabetes specialist nurse, or a comparator group who will receive counselling with a diabetes specialist nurse only. The primary outcomes are device-measured total sitting time and total number of steps (activPAL3). The secondary outcomes are fatigue, health-related quality of life and musculoskeletal problems (self-reported questionnaires), number of sick leave days (diaries), diabetes medications (clinical record review) and cardiometabolic biomarkers including waist circumference, mean blood pressure, HbA1c, HDL-cholesterol and triglycerides.

**Discussion:**

Successful interventions to increase physical activity among those with T2DM have been costly and long-term effectiveness remains uncertain. The use of mHealth technologies such as activity trackers and SMS text reminders may increase awareness of prolonged sedentary behaviour and encourage increase in regular physical activity. mHealth may, therefore, provide a valuable and novel tool to improve health outcomes and clinical management in those with T2DM. This 12-month RCT will evaluate longer-term effects of a mHealth intervention suitable for real-world primary health care settings.

**Trial registration:**

ClinicalTrials.gov NCT04219800. Registered on 7 January 2020.

**Supplementary Information:**

The online version contains supplementary material available at 10.1186/s13063-022-06528-x.

## Administrative information

Note: the numbers in curly brackets in this protocol refer to SPIRIT checklist item numbers. The order of the items has been modified to group similar items (see http://www.equator-network.org/reporting-guidelines/spirit-2013-statement-defining-standard-protocol-items-for-clinical-trials/).

**Table:** 

Title {1}	Health effects of reduced occupational sedentary behaviour in type 2 diabetes using a mobile health intervention: A study protocol for a 12-month randomized controlled trial – the ROSEBUD study
Trial registration {2a and 2b}.	Trial registration: ClinicalTrials.gov Identifier: NCT04219800. First Posted: January 7, 2020, https://clinicaltrials.gov/ct2/show/NCT04219800?term=2019-05383&draw=2&rank=1
Protocol version {3}	2021/02/22, version 1
Funding {4}	The Swedish Diabetes Foundation, Skandia and the Northern county council's regional federation.
Author details {5a}	MB Syrjälä^1^, L Bennet ^2,3,4^, PC Dempsey^5,6,7^, E Fharm^1^, M Hellgren^8^, S Jansson^9,10^, S Nilsson^11^, M Nordendahl^1^, O Rolandsson^1^, K Rådholm^11,12^, A Ugarph-Morawski^13,14^, P Wändell^14^, P Wennberg^1^ ^1^Department of Public Health and Clinical Medicine, Family Medicine, Umeå University, Sweden ^2^ Department of Clinical Sciences, Lund University, Malmö, Sweden ^3^ Center for Primary Health Care Research, Region Skåne and Lund University, Malmö, Sweden ^4^ Baker Heart and Diabetes Institute, Melbourne, Australia ^5^ MRC Epidemiology Unit, Institute of Metabolic Science, University of Cambridge, United Kingdom ^6^ Diabetes Research Centre, University of Leicester, Leicester General Hospital, Leicester, UK ^7^ School of Medical Sciences, University Health Care Research Center, Örebro University, Örebro, Sweden ^8^Skaraborg Institute, Skövde, Sweden ^9^School of Medical Sciences, University Health Care Research Center, Örebro University, Örebro, Sweden ^10^Department of Public Health and Caring Sciences, Uppsala University, Uppsala, Sweden ^11^Department of Health, Medicine and Caring Sciences, Linköping University, Linköping, Sweden ^12^ The George Institute for Global Health, University of New South Wales, Sydney, Australia ^13^Academic Primary Care Center, Region Stockholm^,^ ^14^Department of Neurobiology, Care Sciences, and Society, Division of Family Medicine and Primary Care, The Karolinska Institute, Huddinge
Name and contact information for the trial sponsor {5b}	The Swedish Diabetes Foundation, Skandia and the Northern county council's regional federation.
Role of sponsor {5c}	The funders had no influence on the research reported in this paper.

## Introduction

### Background and rationale {6a}

In Sweden, the prevalence of type 2 diabetes mellitus (T2DM) is increasing in all age groups, except for the youngest [1]. T2DM may not only lead to fatigue and impaired quality of life, but also increases the risk of cardiovascular disease and premature death [2]. Regular physical activity (PA) is an established cornerstone in the management of T2DM [3]. Furthermore, evidence also suggests that reducing and breaking up prolonged sitting time, even with small volumes or bouts of daily low-intensity PA, may provide health benefits in individuals with T2DM [4–6]. However, only one-third of individuals with T2DM in Sweden meet the recommended guidelines for physical activity [7]. The low adherence to PA recommendations highlights the need for alternative strategies.

A recent narrative review suggested that a stepwise PA counselling approach that starts with targeting sedentary behaviour may build a pathway to a more active lifestyle [8]. Moreover, experimental evidence indicates that reducing and breaking up sitting time may be a useful strategy to improve glucose control [5, 6, 9, 10] and quality of life [11], as well as reducing fatigue [12]. Therefore, interrupting prolonged sitting with more light-intensity activities has been suggested as a beneficial and pragmatic intervention for individuals with T2DM, particularly in those who are physically inactive, are obese or have reduced exercise tolerance [13, 14]. Accordingly, the 2016 Position Statement on physical activity/exercise and diabetes from the American Diabetes Association (ADA) includes specific recommendations to reduce and interrupt prolonged sitting [3].

Interventions targeting specific workplaces have been designed to reduce occupational sedentary behaviour in order to reduce the risk of chronic diseases [15]. Pereira et al. found that a multilevel workplace intervention, including the use of sit-stand workstations, is effective for reducing sitting time over a period of 12 months [16]. Further, among participants with prediabetes or diabetes, a meaningful improvement in the clustered metabolic risk scores was noted [16]. However, there is still a paucity of evidence on workplace sedentary interventions targeting individuals with chronic diseases such as T2DM [3, 15, 17]. Furthermore, a 3-year trial showed that individual, biweekly counselling decreased sedentary time [18], but it may not be suitable for implementation in routine primary health care due to resource and personnel constraints.

Mobile health technologies (mHealth) could be used to support reduction in sedentary behaviour and to increase PA at low cost [19].The Global Observatory for eHealth (GOe) has defined mHealth as a medical and public health practice supported by mobile devices, such as mobile phones, patient monitoring and other wireless devices, and personal digital assistants [20]. Activity trackers often include functions that support individual goal setting and self-monitoring, which may enhance motivation and self-efficacy [21]. Importantly, some wrist-worn activity trackers alert the individual after a long bout of uninterrupted sitting and may help to reduce sitting time [19]. Short Message System (SMS) reminders may also facilitate behaviour change [20, 22, 23]. Person-centred care is a common feature of primary health care and involves tailoring treatment strategies to suit the individual [24]. Both activity tracker and SMS text reminders provide individual-level support and may lead to increased awareness of sedentary behaviour in people with T2DM [25].

Despite the wide range of mHealth products and services, this technology remains poorly studied, especially in those with chronic diseases such as T2DM. We recently conducted a 3-month pilot study that included 15 participants with T2DM. This pilot study showed that a mHealth intervention was feasible at several different workplaces [25]. The mHealth intervention included counselling to reduce occupational sitting (by introducing daily activity breaks), an activity tracker to alert the individual of prolonged periods of sitting and SMS text reminders to prompt increase in PA breaks [6]. The results of the qualitative analysis highlighted the importance of individually tailored approaches when aiming to stimulate changes in workplace sitting among people with T2DM.

## Objectives {7}

The aim of this study is to evaluate the effects of a mHealth intervention on sedentary behaviour, PA and health in adults with T2DM in a primary health care setting.

**Fig. 1:**
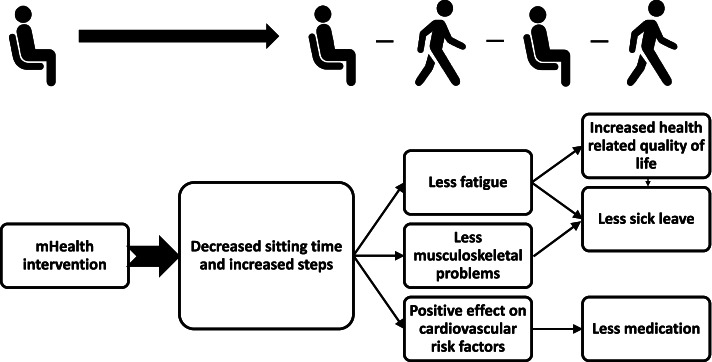
Theoretical model of hypothetical effects of mHealth intervention targeting occupational sitting

The hypothesis is that a mHealth multicomponent intervention will reduce total sitting time and/or increased steps (primary outcome measures) in individuals with T2DM and sedentary occupations. The mHealth intervention will also improve health-related quality of life and reduce cardiometabolic risk, fatigue, sick leave, medication usage and musculoskeletal problems (secondary outcome measures) (Fig. 1).

## Trial design {8}

**Fig. 2:**
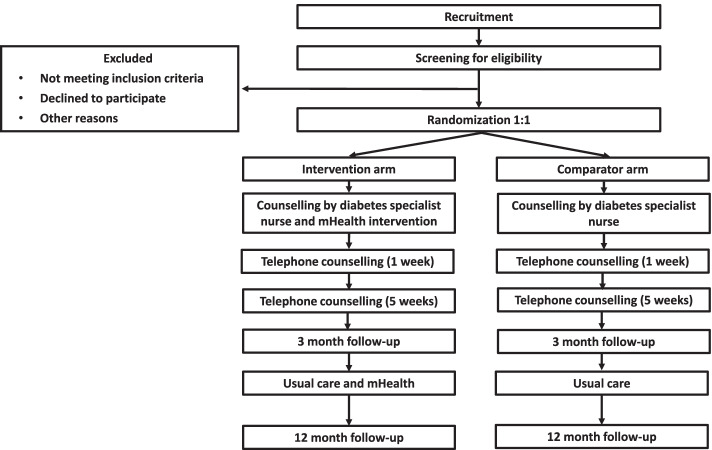
CONSORT flow diagram for the ROSEBUD trial

The ROSEBUD (Reduced Occupational SEdentary BehavioUr in type 2 Diabetes) study is a 12-month dual-arm, superiority randomized controlled trial. Participants will be randomized (1:1) to (a) an intervention arm including mHealth (activity tracker wristband and regular SMS text message reminders) and counselling with a diabetes specialist nurse or (b) a comparator arm including counselling with a diabetes specialist nurse only (Fig. 2). The study will be conducted within a Swedish collaboration for primary health care research in accordance with the CONSORT guidelines and follow the CONSORT EHEALTH Checklist [26, 27].

During the planning of the trial, a qualitative pilot study was conducted to explore the feasibility and acceptability of the mHealth intervention aiming to reduce occupational sitting in individuals with T2DM [25]. The results from the pilot study were used in the final design of the intervention and to determine the sample size of the full-scale study [25].

## Methods: participants, interventions and outcomes

### Study setting {9}

Recruitment and screening of participants will be conducted across the primary health care centres in five regions in Sweden: Norrbotten, Västra Götaland, Östergötland, Stockholm and Örebro. Details on study sites can also be found in ClinicalTrials.gov Identifier: NCT04219800, 2nd of Jan, 2020.

### Eligibility criteria {10}

This study will focus on individuals at an increased risk for diabetes complications. Inclusion criteria are diagnosed with T2DM, aged 40–64 years, BMI ≥ 25 kg/m^2^, HbA1c 53–100 mmol/mol (7.2–11.3%) and working at least 75% in a mainly sedentary occupation (performing > 50% of duties while sitting). Exclusion criteria are pregnancy, regular high-intensity physical training more than 75 min/week, severe immobility or other obstacles to complete the protocol.

### Who will take informed consent? {26a}

The regional coordinating investigator or his/her designee will give eligible participants a detailed oral and written description of the study. An opportunity to ask questions will be provided before the written informed consent is obtained.

### Additional consent provisions for collection and use of participant data and biological specimens {26b}

There are no planned ancillary studies involving the collection or derivation of data for purposes that are separate from the main trial. No additional biological samples will be obtained to be stored for use in future studies.

### Interventions

#### Explanation for the choice of comparators {6b}

We will evaluate whether mHealth (activity tracker and SMS-reminders) reduces sitting time, increases steps and provides health benefits compared to counselling and telephone follow-up by a diabetes specialist nurse alone.

#### Intervention description {11a}

##### Intervention arm

The intervention will begin with individual face-to-face counselling with a diabetes specialist nurse who will use a patient-centred approach focused on occupational sitting [24, 28]. Results from the baseline 1-week activity measurements with thigh-worn accelerometer (activPAL3) will be used for reflection over daily activity patterns. Individual strategies to reduce occupational sitting and stepwise goal setting will be discussed and written down on a goal sheet [21, 29, 30]. Participants will receive written and oral instructions to interrupt prolonged sitting. Interruptions will be gradually increased to 3 min every 30 min of either simple resistance activities or low intensity walking [6]. Telephone follow-ups take place after 1 and 5 weeks. Participants will also receive additional support for mobile health technology. They will use an activity tracker wristband, Garmin Vivofit4 (Nordic Garmin Sweden AB Billdal) that prompts the participant when he/she has been continuously inactive for more than 1 h. Visually, a red ‘move bar’, and auditory, delivers a low short beep; prompts will be received [21]. To reset the ‘move bar’, participants need to walk for a couple of minutes. The activity tracker automatically creates a step goal based on previous activity levels and informs the user when the daily goal is reached. Participants will also receive regular SMS text reminders on the workdays, either daily or weekly depending on personal choice. SMS text reminders will include a link to a mobile video instruction for simple resistance activities [25].

##### Comparator arm

Participants in the comparator arm will only receive individual face-to-face counselling and telephone counselling with a diabetes specialist nurse who will use a patient-centred approach focused on occupational sitting [13, 18].

#### Criteria for discontinuing or modifying allocated interventions {11b}

The participants can withdraw from the study at any time.

#### Strategies to improve adherence to interventions {11c}

**Fig. 3:**
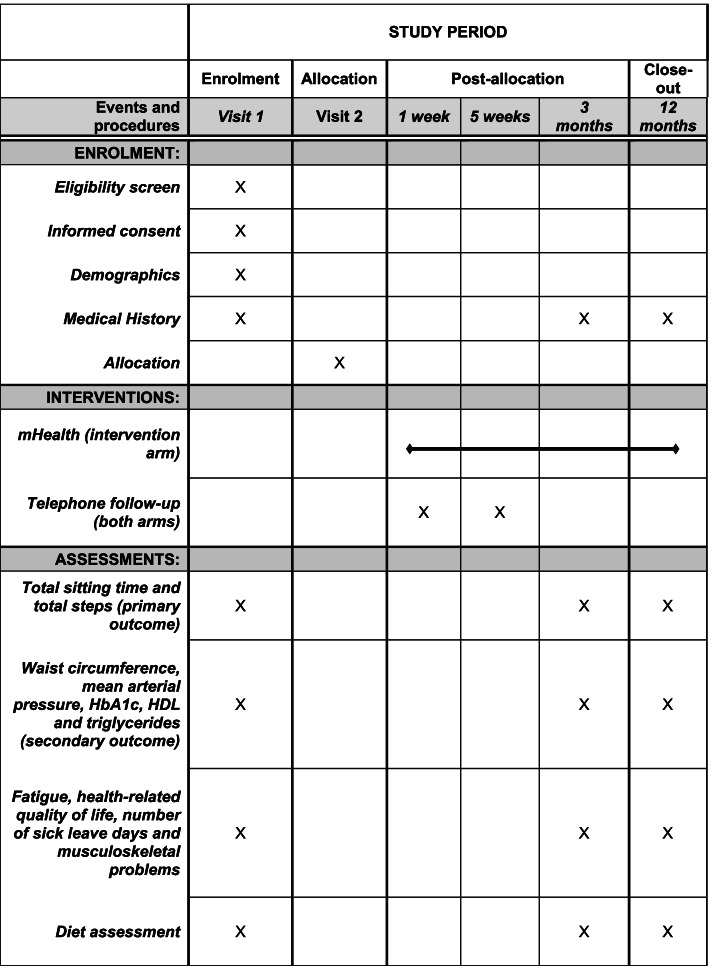
The participant timeline for ROSEBUD trial

During the trial period, all the study participants will have individual face-to-face appointments with the diabetes specialist nurse at 0, 3 and 12 months (Fig. 3). To improve adherence to the protocol, telephone follow-ups will occur 1 and 5 weeks after the face-to-face appointment. Participants in the intervention arm will also receive regular SMS text reminders. At the end of the study, all participants will be asked to respond to a survey with questions concerning barriers and facilitators for reducing occupational sitting.

#### Relevant concomitant care permitted or prohibited during the trial {11d}

Study participation does not replace the usual diabetes care. The usual care in Sweden often involves a multidisciplinary team approach and comprises care by the primary care practitioners, diabetes specialist nurses and nutritionists and ophthalmologists when required.

#### Provisions for post-trial care {30}

There will be no provisions after this trial.

### Outcomes {12}

All primary and secondary outcomes will be measured at study visits at three time points: 0, 3 and 12 months (see also Participant timeline Fig. 3).

#### Primary outcome

Increased physical activity at work may lead to increased sitting time or fewer steps outside working hours [31]. To account for such possible compensatory effects of the intervention, *total* sitting time (minutes per week) was chosen as the primary outcome.

#### Secondary outcome

Secondary outcomes are total steps, fatigue, health-related quality of life, cardiometabolic risk, number of sick leave days, medications and musculoskeletal problems.

#### Other outcomes

Occupational sitting time (minutes per week) and occupational steps (steps per week) will be evaluated in a secondary analysis.

Self-efficacy will be evaluated as an effect modifier of changes in sitting time and steps.

### Participant timeline {13}

The participant timeline for ROSEBUD trial is shown in Fig. 3.

### Sample size {14}

The sample size was calculated using data from a mHealth intervention pilot study (15 participants) [25]. To facilitate the application of the concept minimal clinically important difference (MCID), the sample size was calculated based on results for daily step counts rather than for total sitting time for which MCID has not been established in previous research. Based on an increase of 1496 (standard deviation = 2930) steps/day after 3 months, and incorporating a dropout rate of 15%, a total of 142 participants would have 80% power in finding a statistically significant difference in step count between the groups. Approximately 1500 steps/day can be translated to additional 15–25 min of daily walking (depending on walking speed) which we deem to be a minimal clinically important difference for physically inactive T2DM patients [32].

### Recruitment {15}

The recruitment strategy will include (a) personal communication at diabetes check-ups with diabetes specialist nurses, (b) written invitation and/or invitation via telephone to potential participants using local diabetes patient registers or (c) through advertisement using, e.g. posters and media adverts.

## Assignment of interventions: allocation

### Sequence generation {16a}

Participants are block randomized by a computer-generated sequence (to counteract the imbalance between the centre and skewed sex ratio) into either the intervention or the comparator arm. Randomization will be done after baseline measurements at a ratio of 1:1.

### Concealment mechanism {16b}

Participants will be randomized using REDCap (Research Electronic Data Capture), which include an online, central randomization service. Allocation concealment will be ensured, as the service will not release the randomization code until the participant has been recruited into the trial, which takes place after all baseline measurements have been completed.

### Implementation {16c}

After both participants and employers have given written informed consent. Participants are registered in REDCap (Research Electronic Data Capture). REDCap creates a study identification number for all the participants. The allocation sequence will be generated electronically in blocks to achieve a balance between the intervention and comparator arm at each study centre.

## Assignment of interventions: blinding

### Who will be blinded {17a}

Researchers responsible for statistical analysis will be blinded with regard to the group allocations. The diabetes specialist nurse and laboratory staff will not be blinded.

### Procedure for unblinding if needed {17b}

This is an open-label trial.

## Data collection and management

### Plans for assessment and collection of outcomes {18a}

#### Demographic data

Information on age, sex, country of birth, education, marital status, occupation and tasks assignment/duties, current smoking status, working hours and alcohol consumption will be self-reported. Total use of medications and eventual changes in medications will be collected from the patient records as well as a participant diary during the study period. Diabetes complications and duration as well as other chronic diseases are collected from the patient records. Participants keep diary on sick leave days during the study period.

#### Sedentary time and steps

Total and occupational sitting time (minutes per week) and total steps (steps per week) will be measured with a thigh-worn, activPAL3™ accelerometer (PAL Technologies Limited, Glasgow, UK). The accelerometer will be worn for 24 h per day for 7 consecutive days [22]. The device is initialized using manufacturer’s software with the default settings (i.e. 20 Hz, 10 s minimum sitting-upright period) and will be covered in a nitrile sleeve and fully wrapped in waterproof dressing to allow participants to wear the device during bathing activities. Participants will keep a diary on their working days and working hours.

#### Biochemical variables

HbA1c and lipids (serum total cholesterol, HDL cholesterol and triglycerides) will be assessed using standard laboratory methodology.

The health benefits of reduced sedentary behaviour are likely mediated by several mechanisms. The results will, therefore, be weighed together in a clustered cardiometabolic risk score (CCMR) [33]. CCMR will be determined by summing *z*-scores ([value − mean]/standard deviation (SD)) of waist circumference (cm), mean arterial pressure (mmHg), HbA1c (mmol/mol), the inverse of HDL (mmol/L) and triglycerides (mmol/L), using sex-specific means and SDs based on previous studies [33, 34]. The use of a common mean and SD for standardized variables at two time points ensures that changes in the score can vary from zero. We will divide both by 5, separately, to account for the number of variables included. Change in the CCMR will be calculated by subtracting the follow-up CCMR from the baseline CCMR. Mean arterial pressure provides a better representation of the average pressure throughout a single cardiac cycle and will be used instead of systolic and diastolic blood pressure. Mean arterial pressure will be calculated using the formula (systolic pressure + [2 × diastolic pressure]/3) described as being one third of the distance between the systolic pressure and diastolic pressure [34].

#### Anthropometric data

Height and weight are measured without shoes, to the nearest 0.1 kg for body weight and to the nearest 0.1 cm for height. BMI is calculated as weight (kg)/height (meters^2^). The blood pressure will be measured after a 5–10-min rest, sitting on the chair with both feet on the floor and always in the same arm. The average of two readings are recorded for systolic and diastolic blood pressure. Waist circumference is measured in a standing position with a tape measure to the nearest 0.5 cm at the level midway between the lower rib margin and the iliac crest during a light exhale. Waist circumference is measured twice, and if there is a difference > 1 cm, the measurements are repeated; otherwise, a mean value is counted to the nearest 0.5 cm. The same measuring tape will be used during the whole study period.

#### Health-related quality of life

Health-related quality of life will be measured by the RAND-36 [25]. The Swedish translation of the RAND-36 is conceptually equivalent to the English version [26].

#### Fatigue

Fatigue will be measured using the four-item General Fatigue subscale of the Multidimensional Fatigue Inventory-20 (MFI-20) [27]. MFI-20 is a self-reporting and validated test, incl. the Swedish version [28]. MFI-20 is a 20-item multidimensional tool that uses a Likert-type scale from 1 to 5, with total scores with range 4–20. MFI-20 contains 5 subscales: General Fatigue, Physical Fatigue, Reduced Motivation, Reduced Activity and Mental Fatigue [27].

#### Musculoskeletal problems

A numerical rating scale (NRS) questionnaire will be used to measure pain intensity [29]. The scale has 11-points (i.e. 0–10) with 0 meaning ‘No pain’ and ‘10’ meaning ‘Pain as bad as you can imagine’, accompanied by the instructions ‘Please rate your pain by indicating the number that best describes your pain on average in the last 24 h is recommended as a core outcome measure in clinical trials of chronic pain treatments’.

#### Self-efficacy

Swedish translation of General Self-Efficacy scale (GSE-10) is used to assess the strength of an individual’s belief in his/her own ability to respond to difficult situations and to deal with setbacks [30].

#### Diet assessment

During the activity measurement week, the participants will keep a food diary of everything they eat and drink (excl. water) during three consecutive days (including one weekend day; Saturday or Sunday). The results are compiled using a programme for nutritional calculations (Dietist Net Pro).

### Plans to promote participant retention and complete follow-up {18b}

Participants are provided with a study calendar as a reminder of upcoming appointments and follow-up phone calls to promote participant retention and completion.

### Data management {19}

Study data will be collected and managed using REDCap electronic data capture tools hosted at Umeå University. REDCap is a secure, web-based software platform designed to support data capture for research studies. REDCap provides (1) an intuitive interface for validated data capture, (2) audit trails for tracking data manipulation and export procedures, (3) automated export procedures for seamless data downloads to common statistical packages and (4) procedures for data integration and interoperability with external sources [35, 36].

REDCap uses Electronical Case Report Forms (eCRF) which increases the compliance to the study protocol used by diabetes specialist nurse. REDCap directly notifies if any data is missing. The programme allows secure file uploading of activPAL data as well as records/diaries.

Scheduling function in eCRF makes it possible to schedule the telephone follow-up and if data is lacking, i.e. when it is time to make a follow-up telephone call and if participant is receiving the SMS text messages as planned.

### Confidentiality {27}

REDCap software ensures the secure data collection and maintenance both during and after the trial.

### Plans for collection, laboratory evaluation and storage of biological specimens for genetic or molecular analysis in this trial/future use {33}

No biological specimens obtained during the conduct of the trial will be stored for future use.

## Statistical methods

### Statistical methods for primary and secondary outcomes {20a}

Random effects mixed model analysis will be used to determine changes between the two study arms. The threshold for statistical significance will be set at *p* < 0.05. To avoid overestimating the impact of the intervention, an intention-to-treat approach will be adopted to evaluate the impact on the primary and secondary outcomes.

### Interim analyses {21b}

No interim analyses will be conducted as we do not foresee any potentially serious outcomes.

### Methods for additional analyses (e.g. subgroup analyses) {20b}

Self-efficacy is considered a potential moderator for behavioural interventions [21]. Therefore, analyses will be stratified based on self-efficacy level in a subgroup analysis.

### Methods in analysis to handle protocol non-adherence and any statistical methods to handle missing data {20c}

By using a random effects mixed model, all participants with at least one valid measurement will be included in the analysis. Hence, intermittent missing is not expected to substantially bias the results. To address possible informative drop-outs, a sensitivity analysis will be performed using multiple imputation, including all available information on background characteristics and outcome. Random effects mixed model analysis will be used to handle data missing at random for any of the two follow-ups [37].

### Plans to give access to the full protocol, participant-level data and statistical code {31c}

There are no plans for granting public access to the full protocol, participant-level data and statistical code.

## Oversight and monitoring

### Composition of the coordinating centre and trial steering committee {5d}

The coordinating centre consists of the principal coordinating investigator (PW) and a PhD student (MBS) who support in the coordination of the project. The trial steering committee consists of the regional coordinating investigators (MH, SJ, MN, KR and AU-M) and is led by the principal coordinating investigator.

### Composition of the data monitoring committee, its role and reporting structure {21a}

Research coordinators at the clinical research centre at Umeå University/Region Västerbotten and the principal coordinating investigator will regularly monitor data that is entered in REDCap. The clinical research centre is independent from the sponsor and has no competing interests.

### Adverse event reporting and harms {22}

The regional coordinating investigator will be notified by study personnel in case of an adverse event. All adverse events will be reported in REDCap.

### Frequency and plans for auditing trial conduct {23}

Not applicable. There is no on-site auditing of the trial.

### Plans for communicating important protocol amendments to relevant parties (e.g. trial participants, ethical committees) {25}

The trial steering committee will communicate substantial protocol modifications (when applicable) to relevant parties (referral Ethics Committee, trial participants and study personnel).

## Dissemination plans {31a}

Results of the trial will be published in international peer-reviewed scientific journals. There is no obligation for communicating the results to individual patients.

## Discussion

The ROSEBUD study will evaluate whether mHealth (activity tracker and SMS text reminders), in addition to counselling and telephone follow-up by a diabetes specialist nurse, will lead to reduced sitting time and increased steps. In addition, the ROSEBUD study will contribute to an increased understanding of the long-term health impact of occupational sedentary behaviour in adults with T2DM. If effective, this intervention could be implemented within typical primary health care settings and integrated in regular diabetes specialist nurse check-ups.

The modern office workplace is conducive to workers spending large amounts of time sitting [31]. Prolonged sitting time has emerged as a significant health concern which makes the workplace an important target for intervention [38]. The health benefits of more movement and less sedentary time are now well recognized. Indeed, the recent WHO guidelines now also promote reductions in sitting time [39]. However, research on the health effects of breaks in prolonged sitting time in people with chronic conditions like T2DM is still limited [40, 41]. Also, increasing movement at work may be more challenging for people with obesity and T2DM [42]. Nevertheless, previous research suggests that interventions using a variety of strategies are more likely to succeed [5, 32]. Accordingly, interventional strategies that include education on sedentary behaviour, motivational support and environmental cues (i.e. sit-stand desks) may facilitate reduced sitting at work [5, 11, 32]. mHealth technologies, including SMS text reminders and activity trackers, seem to support the individual behaviour change in individuals with T2DM and could therefore be valuable for individualized feedback in several different workplaces [25]. In addition, several wrist-worn activity trackers are programmed to prompt the users to reduce prolonged bouts of uninterrupted sitting [21].

Among those with T2DM, prolonged, uninterrupted sitting increases fatigue compared to interrupted sitting by regular brief activity breaks [4]. A reduction in prolonged occupational sitting may not only reduce fatigue and increase health-related quality of life but may also limit the number of sick days and improve work productivity [43]. Few trials evaluated interventions that reduce occupational sitting time among employees with chronic diseases. The ‘Stand and Move at Work’ multicomponent workplace intervention study tested the effects on health parameters of sit-stand workstations among 630 full-time employees of which 95 participants had prediabetes or diabetes [16]. Among the subgroup with prediabetes and diabetes, even small improvements in physical activity and sedentary time might be associated with a clinically meaningful decrease in cardiovascular risk [16]. A systematic review and meta-analysis found that interventions targeting sedentary behaviour reductions (alone or combined with increases in PA) are effective in decreasing cardiometabolic risk to a small degree but there is still paucity of evidence on long-term interventions in populations with chronic diseases such as T2DM [17].

In conclusion, a cost-effective, pragmatic intervention is needed to reduce the detrimental effects of prolonged uninterrupted sitting among individuals with T2DM. However, long-term trials are limited. Simple and easy-to-use activity trackers and SMS text messages are often inexpensive and therefore realistic to be utilized in primary health care settings. The ROSEBUD study will provide more clarity on the long-term health effects of a mHealth intervention for reduced sitting time in people with T2DM. Further, this study will provide clinically important knowledge that may improve diabetes management.

## Trial status

The first participant was recruited on the 14th of December 2020. The recruitment process is planned to be completed approximately on the 31st of December 2022.

## Supplementary Information

**Additional file1.** Sample informed consent.

## Data Availability

The first and the last author as well as the monitoring committee of the clinical research centre at Umeå University/Region Västerbotten have access to the final trial dataset.

## Abbreviations

T2DM: Type 2 diabetes mellitus
mHealth: Mobile health technology
PA: Physical activity
RCT: Randomized controlled trial
ADA: American Diabetes Association
GOe: The Global Observatory for eHealth
SMS: Short Message System
ROSEBUD: Reduced Occupational SEdentary BehavioUr in type 2 Diabetes
REDCap: Research Electronic Data Capture
eCRF: Electronic Case Report Forms
BMI: Body mass index
HbA1c: Haemoglobin A1c
MFI-20: The Multidimensional Fatigue Inventory-20
GSE-10: General Self-Efficacy scale
