# Supplementary material for: Health effects of reduced occupational sedentary behaviour in type 2 diabetes using a mobile health intervention: a study protocol for a 12-month randomized controlled trial—the ROSEBUD study
Source: Trials. 2022 Jul 27;23:607. doi: 10.1186/s13063-022-06528-x (PMC9331801; doi:10.1186/s13063-022-06528-x)
Supplement: Supplementary file 1 — Additional file1. Sample informed consent. [file 13063_2022_6528_MOESM1_ESM.pdf]

Samtyckesformulär

Jag har fått muntlig och skriftlig information om studien och har haft möjlighet att ställa frågor. Jag får behålla den skriftliga informationen.

☐ Jag samtycker till att delta i studien ”Minska stillasittande på arbetet”.

☐ Jag samtycker till att uppgifter om mig behandlas på det sätt som beskrivs i forskningspersonsinformationen.

☐ Jag samtycker till att bli kontaktad med förfrågan att delta i andra studier inom detta forskningsområde.

Datum: _______________

Underskrift: ____

Namnförtydligande: _______________________________________

Information given av:

Datum: _______________

Underskrift: ____

Namnförtydligande: _______________________________________

Version 2020:5
